# Supplementary material for: Structures of chaperone-substrate complexes docked onto the export gate in a type III secretion system
Source: Nat Commun. 2018 May 2;9:1773. doi: 10.1038/s41467-018-04137-4 (PMC5932034; doi:10.1038/s41467-018-04137-4)
Supplement: Supplementary file 1 — Supplementary Information [file 41467_2018_4137_MOESM1_ESM.pdf]

Supplementary Information for

Structures of chaperone-substrate complexes docked onto the export gate in a type III secretion system

Xing et al.

This file includes:

Supplementary Figures 1-11
Supplementary Tables 1-3

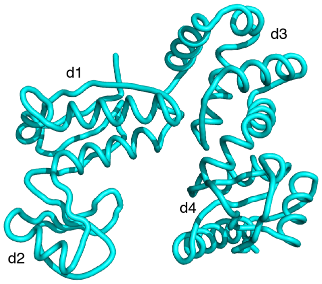

FlhA^{C} *S. enterica* (PDB ID 6CH1)
cleft opening: 12 Å

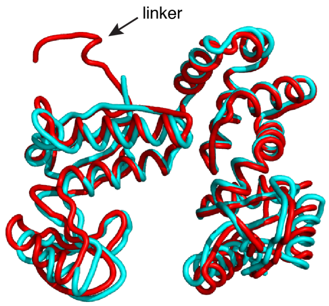

FlhA$^{C\text{-}link}$ *S. enterica* (PDB ID 3A5I)
Z: 41.7, RMSD: 2 Å, Identity: 100%
cleft opening: 16.7 Å

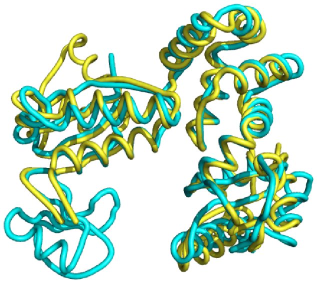

FlhA$^{C\text{-}link}$ *B. subtilis* (PDB ID 3MIX)
Z: 32.2, RMSD: 3.2 Å, Identity: 34%
cleft opening: d2 domain not visible

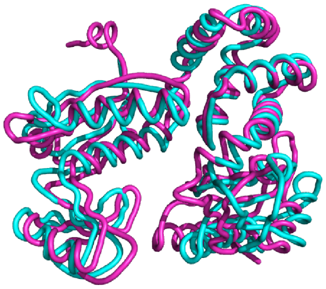

FlhA$^{C\text{-}link}$ *H. pylori* (PDB ID 3MYD)
Z: 32.5, RMSD: 3.7 Å, Identity: 38%
cleft opening: 6.5 Å

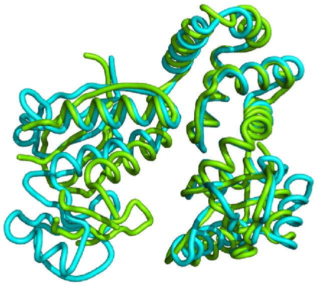

InvA^{C} *S. enterica* (PDB ID 2X49)
Z: 24.2, RMSD: 5.3 Å, Identity: 23%
cleft opening: 5.9 Å

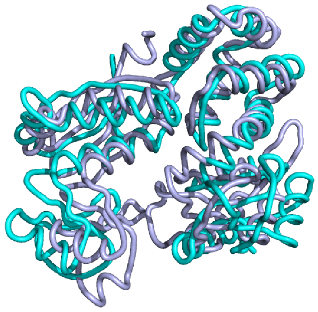

MxiA *S. flexneri* (PDB ID 4A5P)
Z: 25.8, RMSD: 3.8 Å, Identity: 24%
cleft opening: 7.5 Å

Supplementary Figure 1. Structural similarity between FlhA and its homologs.

Superposition of the crystal structure of FlhA^{C} (in cyan) on the structure of FlhA proteins from other species and the homologous proteins InvA and MxiA from pathogenic T3SSs. The Z score, Ca rmsd an identity percentage were calculated by the Dali server (http://ekhidna.biocenter.helsinki.fi/dali_server). Cleft opening was measured as the distance between the nearest residues in the d2 and d4 domains.

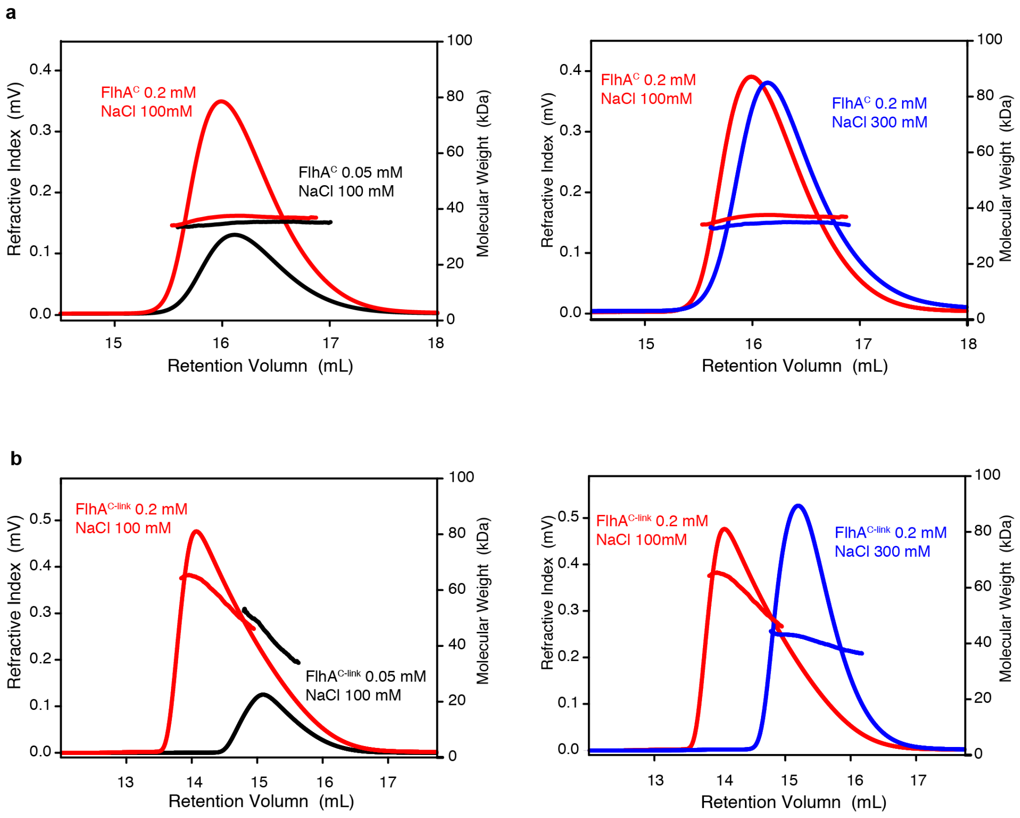

Supplementary Figure 2. Oligomerization of FlhA^{C}.

(a) SEC-MALS profiles of FlhA^{C} in two different protein concentrations and salt concentrations. FlhA^{C} elutes as a monomer. **(b)** SEC-MALS profiles of FlhA$^{C\text{-link}}$ in two different protein concentrations and salt concentrations. FlhA$^{C\text{-link}}$ elutes as a dimer under physiological salt concentrations. Because dimerization is mediated almost exclusively by means of electrostatic interactions, higher salt concentrations disrupt FlhA^{C} dimerization. FlhAC is in fast equilibrium between a monomeric and dimeric state.

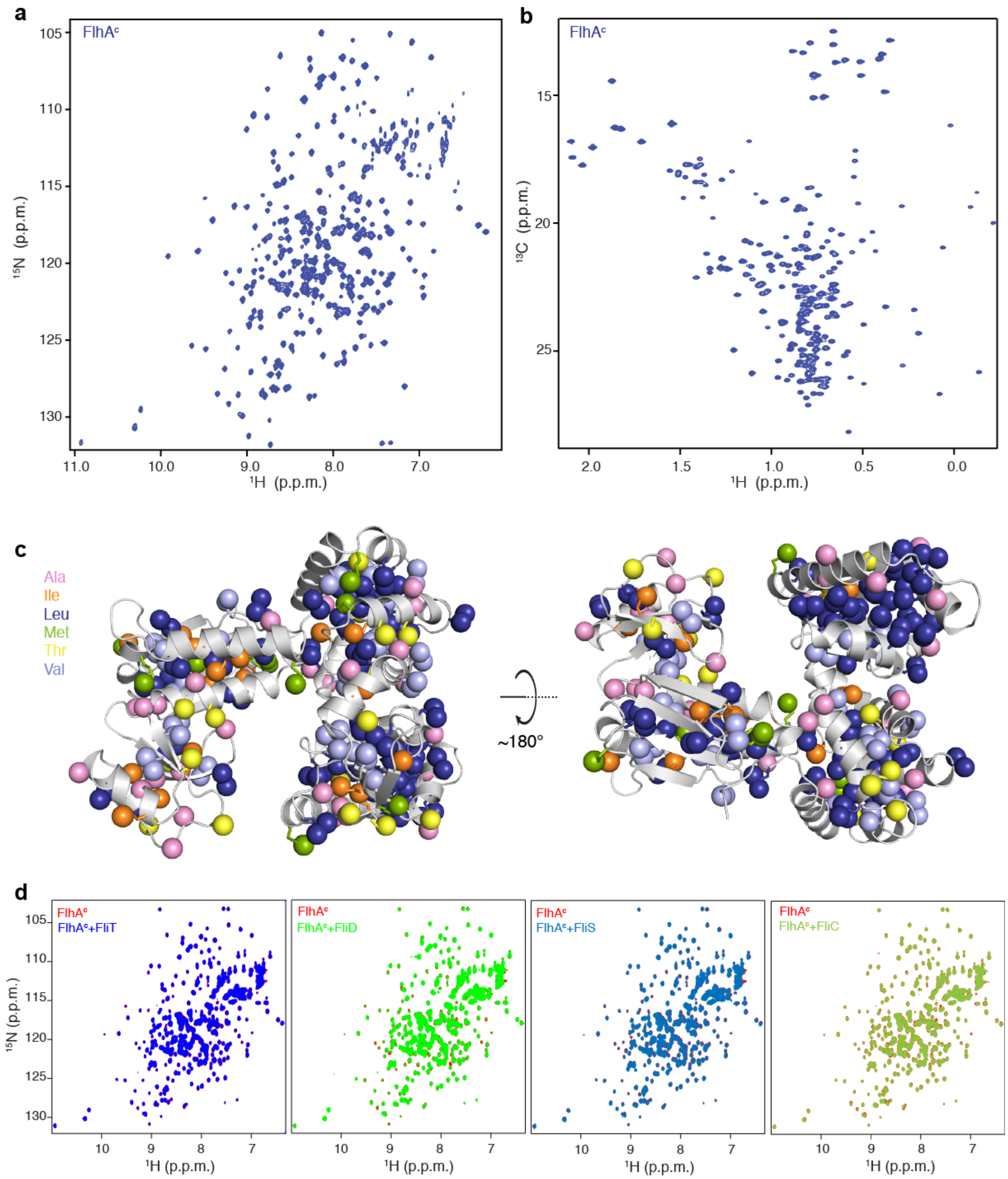

Supplementary Figure 3. NMR characterization of FlhA^C.

(a,b) ^{1}H-^{15}N transverse relaxation-optimized (TROSY) spectra (a) and ^{1}H-^{13}C HMQC spectra (b) of U-^{2}H, Ala- ^{13}CH$_3$, Met-^{13}CH$_3$, Ile-δ1-^{13}CH$_3$, Leu, Val-^{13}CH$_3$ and Thr-^{13}CH$_3$ labeled FlhA^c. **(c)** Distribution of methyl-bearing amino acids in FlhA^C. **(d)** ^{1}H-^{15}N TROSY spectra of FlhA^C (0.4 mM) titrated, at a molar ratio 1:2, with free FliT and FliS chaperones, and free FliD and FliC substrates.

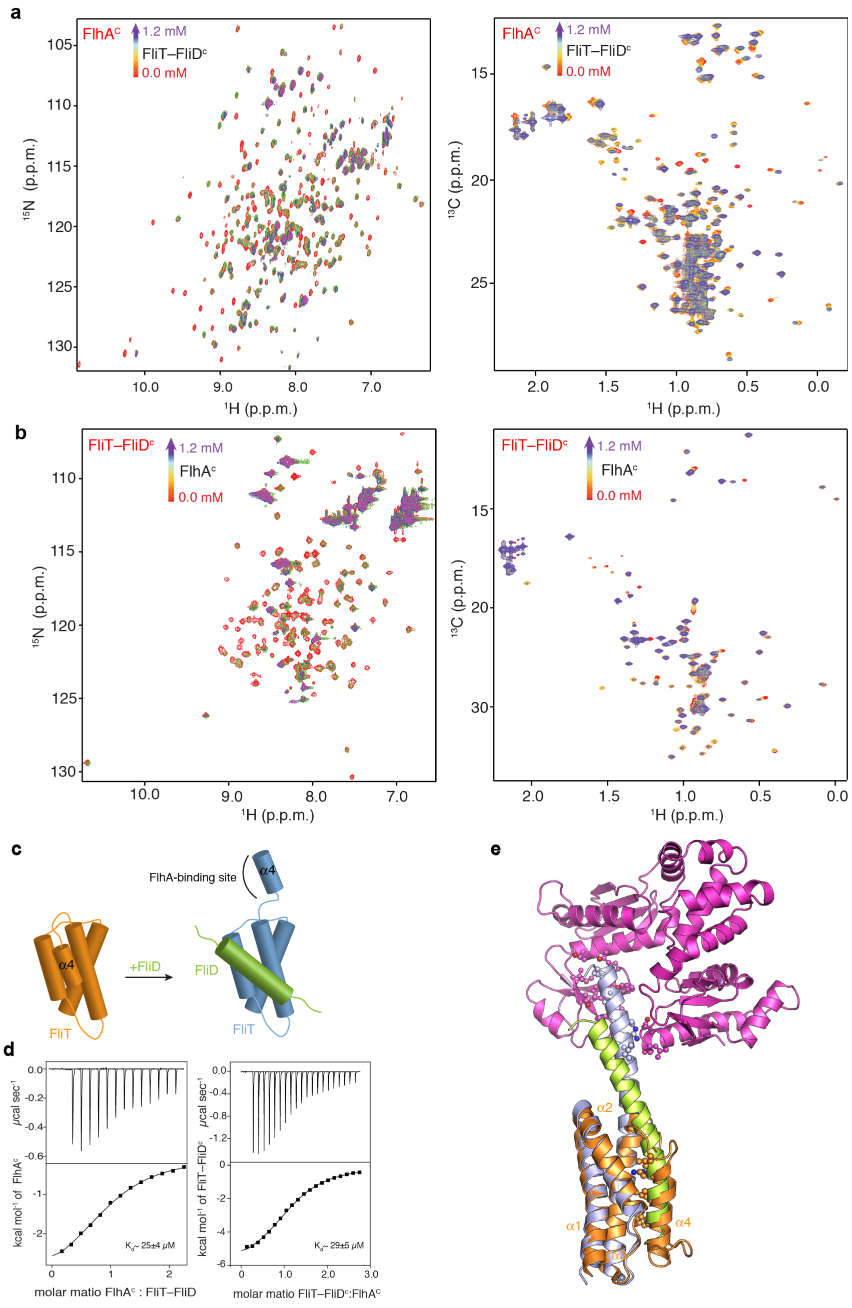

Supplementary Figure 4. Interaction of FlhA^{C} with FliT−FliD.

(a) Titration of unlabeled FliT−FliD^{C} to isotopically labeled FlhA^{C}. The stepwise process was monitored by recording ^{1}H-^{15}N TROSY HSQC and ^{1}H-^{13}C methyl HMQC spectra. **(b)** The reverse titration was similarly monitored by NMR. **(c)** A cartoon depicting how FliD binding to FliT activates it for binding to FlhA by releasing helix α4. **(d)** ITC profiles of FlhA^{C} binding to FliT−FliD, which includes the full-length FliD construct, and FliT−FliD^{C}. The data showed that increasing the length of FliD to encompass additional residues outside the FliT-binding site has no effect on the binding affinity. The K$_{d}$ values are average of a triplicate experiment. **(e)** Superposition of free FliT (orange) on the FliT molecule in the ternary complex (light blue).

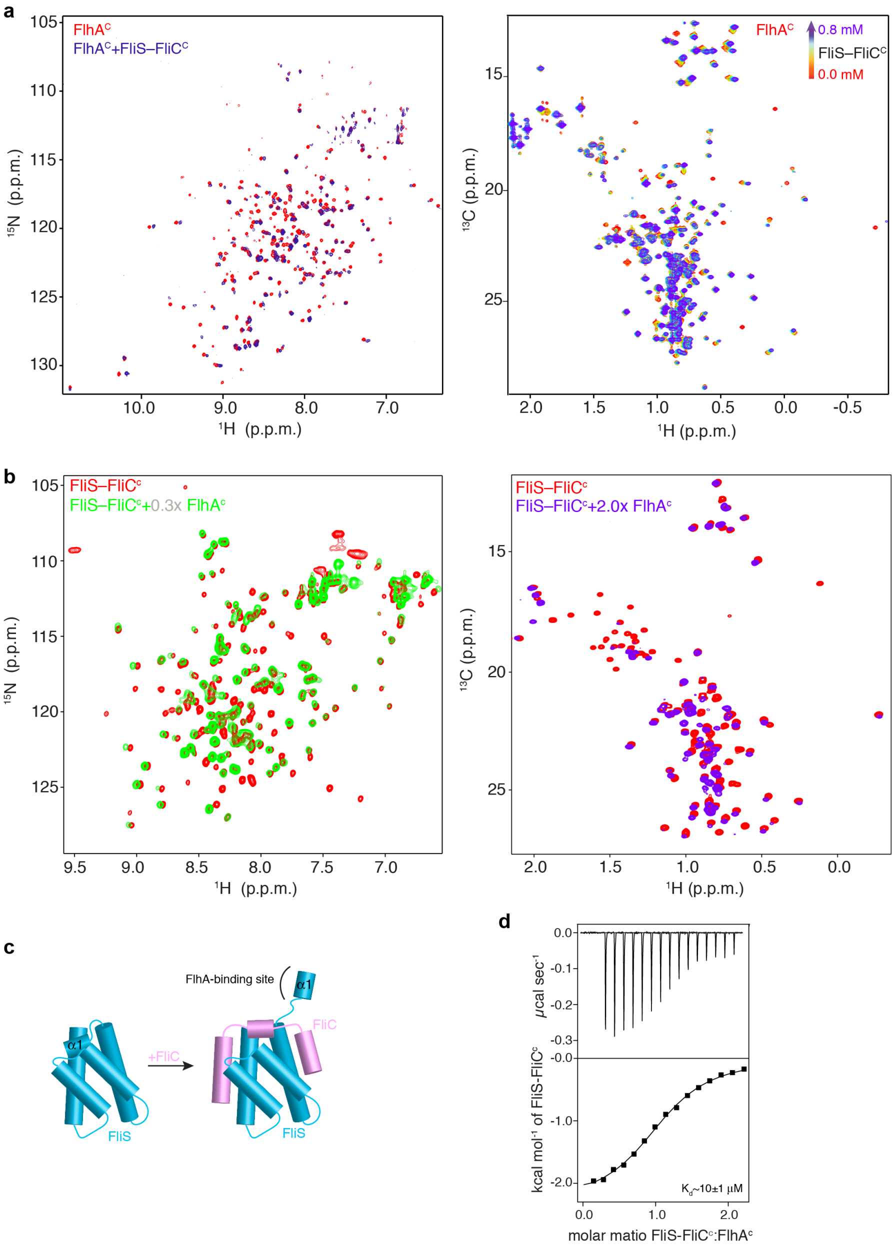

Supplementary Figure 5. Interaction of FlhA^{C} with FliS−FliC.

(a) Titration of unlabeled FliS−FliC^{C} to isotopically labeled FlhA^{C}. The stepwise process was monitored by recording ^{1}H-^{15}N TROSY HSQC and ^{1}H-^{13}C methyl HMQC spectra. **(b)** The reverse titration was similarly monitored by NMR. **(c)** A cartoon depicting how FliC binding to FliS activates it for binding to FlhA by releasing helix α1. **(d)** ITC profile of FlhA^{C} binding to FliS−FliC^{C}. The K_d value is average of a triplicate experiment.

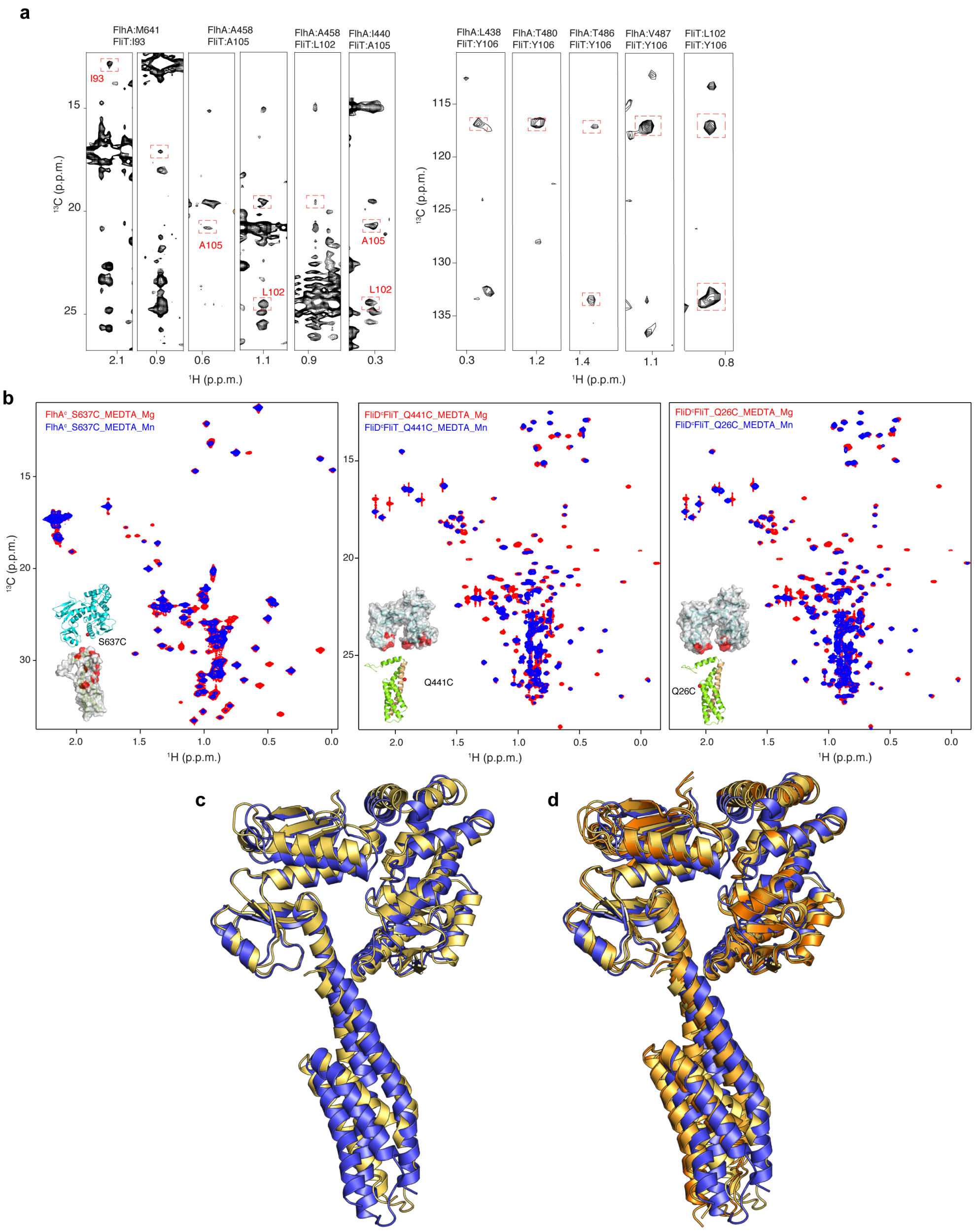

Supplementary Figure 6. NMR structure determination of the FlhA^{C}−FliT−FliD^{C} ternary complex

(a) Representative NOESY strips showing intermolecular NOEs between FlhA^{C} and FliT in the ternary complex. **(b)** ^{1}H-^{13}C methyl HMQC spectra of FlhA^{C} (or FliT−FliD^{C}) bound to FliT−FliD^{C} (or FlhA^{C}) that is conjugated with diamagnetic (Mg^{+2}) or paramagnetic (Mn^{+2}) probe for the measurement of PRE rates in the ternary complex. The single-cysteine positions where the probe was incorporated are shown. **(c)** Superposition of the NMR solution structure (blue) with the crystal structure (light orange), both determined in this work. **(d)** Three FlhA^{C} molecules were present in the asymmetric unit, which are superimposed on the NMR structure. The comparison indicates the variation in the long helices of FliT and FliD located away from the binding site, highlighting the increased flexibility of these helices.

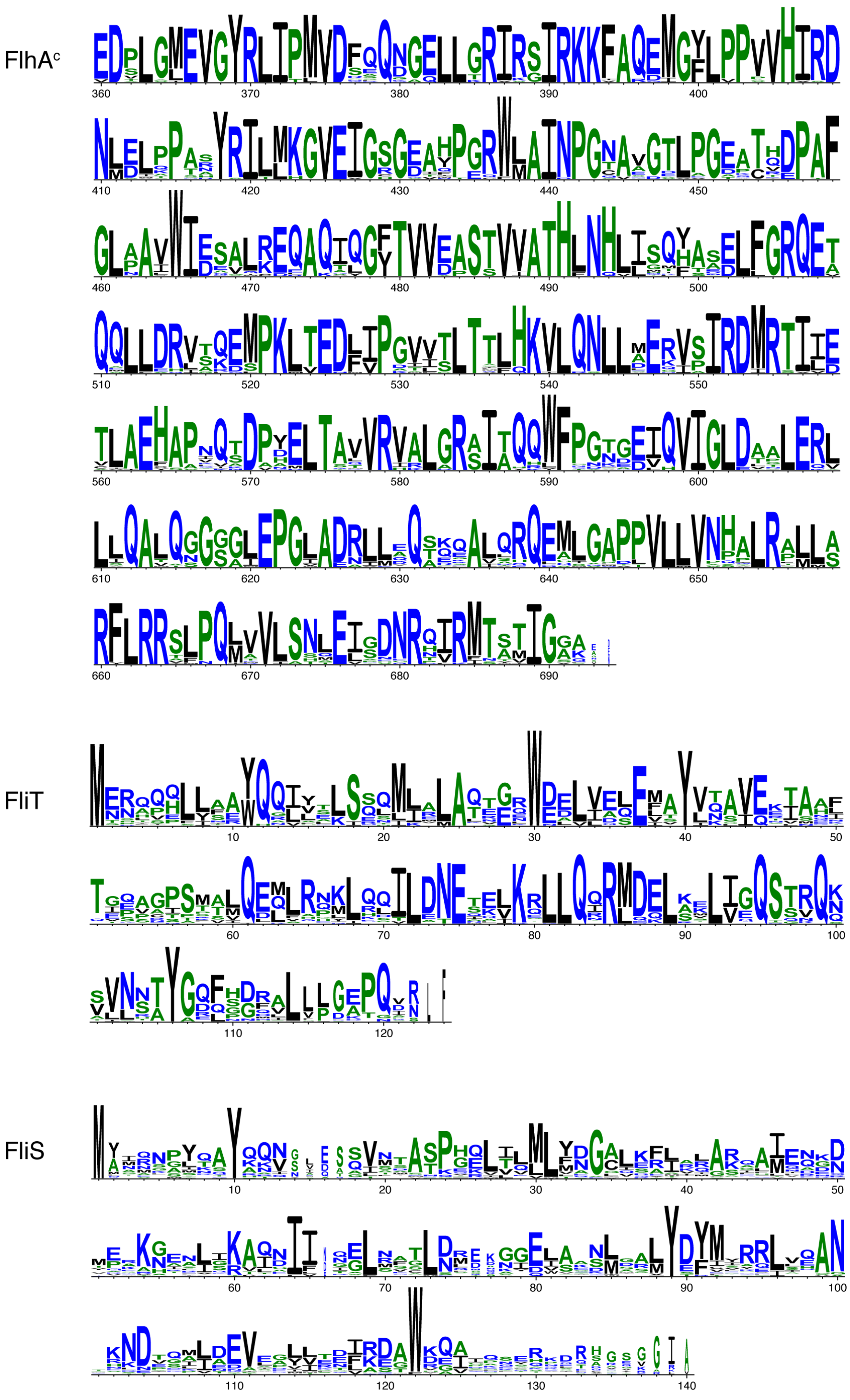

Supplementary Figure 7. Sequence conservation of FlhA^C, FliT and FliS.

Sequence logo of the proteins was created from a collection of aligned sequences. 40 unique sequences were used and the sequence of *Salmonella enterica* is shown.

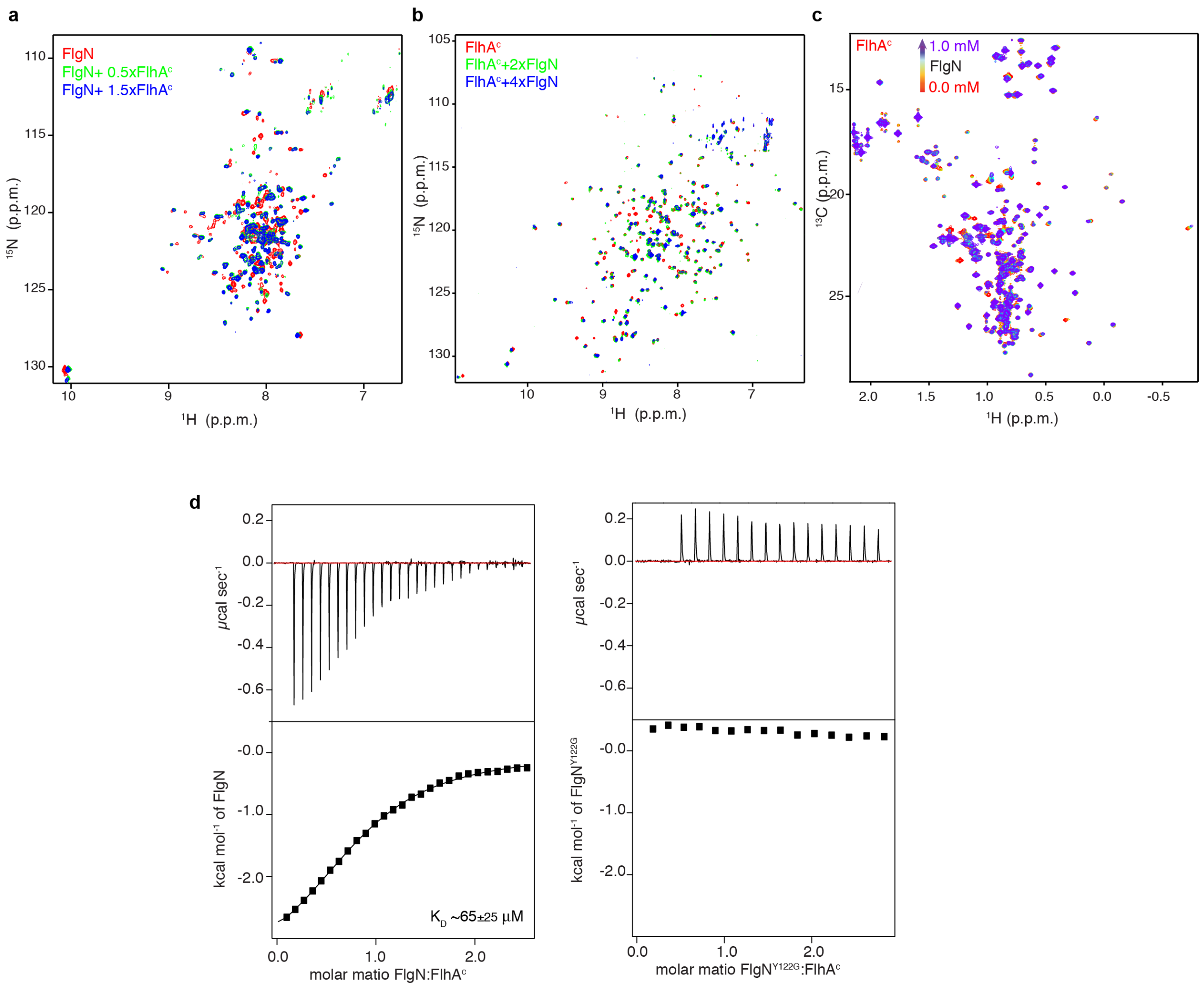

Supplementary Figure 8. Interaction of FlhA^{C} with FlgN.

(a) ^{1}H-^{15}N TROSY HSQC spectra of ^{15}N-labeled FlgN titrated with unlabeled FlhA^{C}. **(b,c)** ^{1}H-^{15}N TROSY HSQC (b) and ^{1}H-^{13}C methyl HMQC (c) spectra of labeled FlhA^{C} titrated with unlabeled FlgN. **(d)** ITC profiles of FlgN (left) and FlgN^{Y122G} (right) binding to FlhA^{C}. FlgN Tyr122 is very important for the formation of the ternary complex and its substitution results in abrogation of the binding. The K$_d$ values are average of a triplicate experiment.

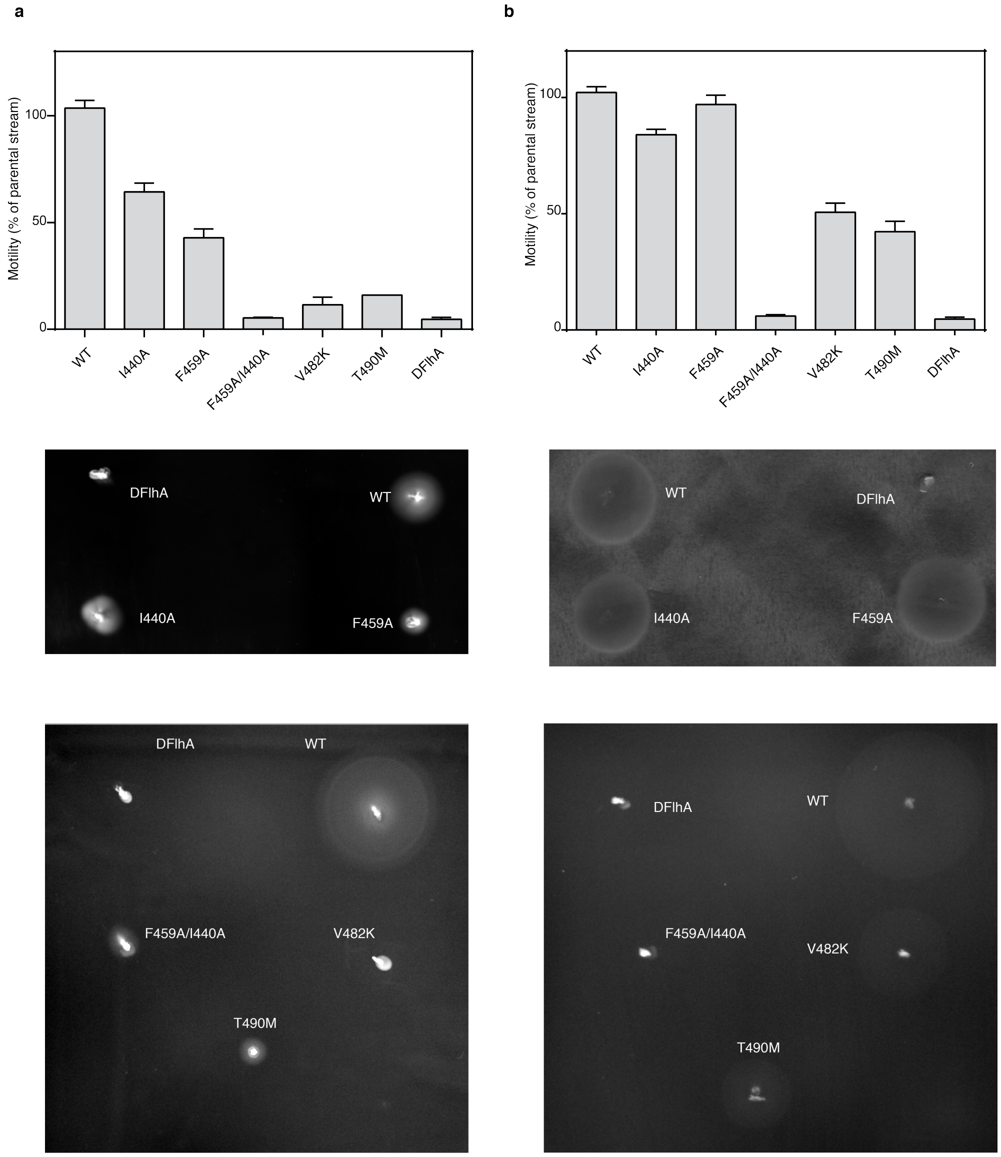

Supplementary Figure 9. Bacterial motility assays

Motility of *Salmonella serovar typhimurium* Δ*flhA* strain (FlhA-) with pkG116 plasmid containing FlhA, $FlhA^{I440A}$, $FlhA^{F459A}$, $FlhA^{F459A/440A}$, $FlhA^{V482K}$ or $FlhA^{T490M}$ in soft agar after incubation at 37 °C, 6 h without inducer (a) or with inducer added (b). FlhA or related FlhA mutant cloned into pkG116 plasmid was overexpressed by the addition of 5 mM IPTG into media. Bar graphs represent the mean value of the colony diameter and error bars represent standard deviation (n=6).

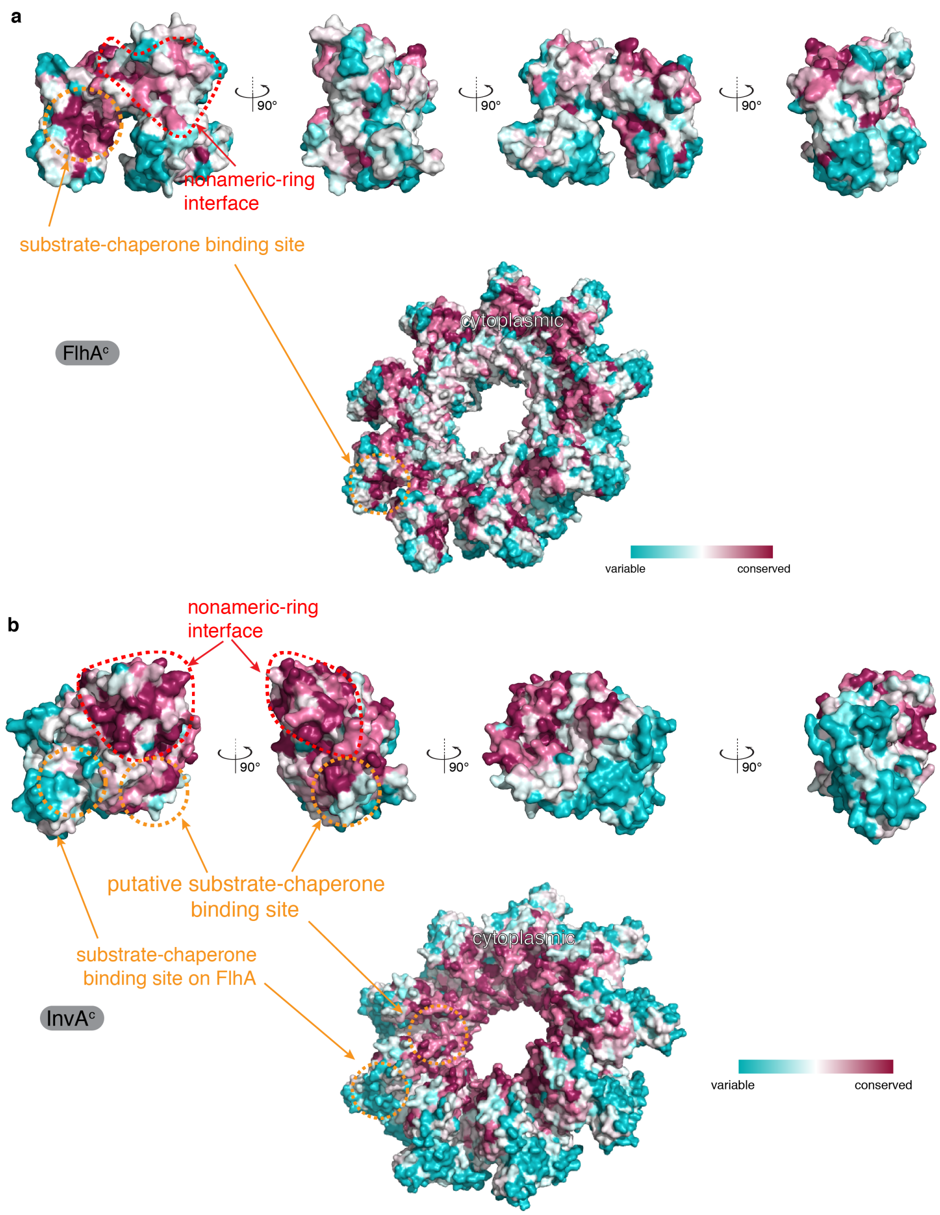

Supplementary Figure 10. Sequence conservation in FlhA^C and SctV^C.

(a) Sequence conservation of FlhA^C colored according to residue identity conservation scores obtained by ConSurf. The oligomeric interface and the identified chaperone-substrate binding site are indicated.

(b) Sequence conservation of SctV^C mapped on the structure of InvA^C from *Salmonella*. The oligomeric interface and a putative chaperone-substrate binding site are indicated.

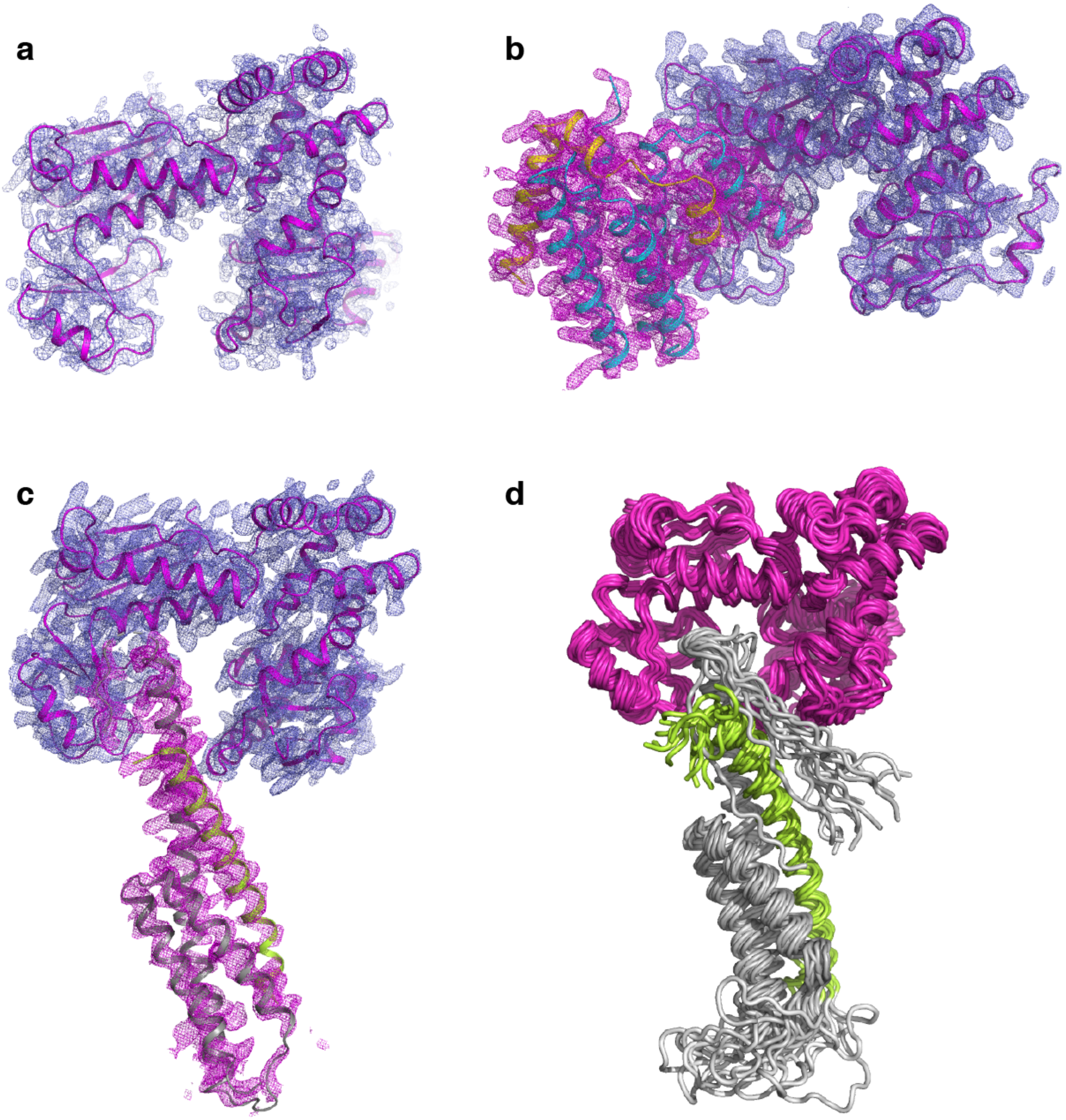

Supplementary Figure 11. Electron density maps and ensemble of the protein structures.

(a) Stereo view of $FlhA^C$ with the 2Fo-Fc electron density map with contour at 1.2 sigma level. **(b)** Stereo view of $FlhA^C$ in complex with FliS-FliC with contour at 1.0 sigma level. **(c)** Stereo view of $FlhA^C$ in complex with FliT-FliD with contour at 1.0 sigma level. **(d)** Ensemble of the 15 lowest-energy NMR structural conformers of $FlhA^C$-FliT-$FliD^C$.

Supplementary Table 1: NMR and refinement statistics for protein structure

	**FlhA^{c}-FliT-FliD**
NMR distance and dihedral constraints	
Total NOE	2017
Intra-molecular	
intra-residue [i = j]	121
sequential [| i - j | = 1]	690
medium range [1 < | i - j | < 5]	686
long range [| i - j | ≥ 5]	1113
Inter-molecular	37
NOE constraints per restrained residue	4.23
Hydrogen bond	298
Total dihedral angle restraints (°)	914
phi	457
psi	457
Intermolecular PRE restraints	690
Total number of restricting constraints	3859
Total number of restricting constraints per restrained residue	8.1
Restricting long-range constraints per restrained residue	2.5
Structure statistics	
Violations (mean ± s.d.)	
Distance constraints (Å)	0.00016±0.00422
Dihedral-angle constraints (°)	0.0038 ± 0.0944
Max.distance-constraint violation (Å)	0.26
Max.dihedral-angle violation (°)	8.23
Deviations from idealized geometry	
Bond lengths (Å)	0.02
Bond angles (°)	1.2
Impropers (°)	0
Average pairwise r.m.s. deviation (Å)[a]	
Heavy	1.8
Backbone	1.5

NOE, nuclear Overhauser effect; PRE, paramagnetic relaxation enhancement; s.d., standard deviation; r.m.s.d., root mean squared deviation.
[a]Pairwise r.m.s. deviation was calculated among 20 refined structures.

Supplementary Table 2. X-ray Data collection and refinement statistics for protein structures.

	FlhA^c	FlhA^c-FliT-FliD	FlhA^c-FliS-FliC
Data collection			
Wavelength	0.979	0.979	0.979
Resolution range (Å)	19.91 - 1.90<br>(1.97 - 1.90)	48.38 - 2.75<br>(2.85 - 2.75)	58.66 - 2.62<br>(2.71 - 2.62)
Space group	P1	P1	$P2_12_12_1$
Cell parameters			
a, b, c (Å)	40.38 49.97 50.95	49.11 77.59 119.27	73.30 88.62 117.33
α, β, γ (º)	78.04 68.99 80.66	86.88 89 84.27	90 90 90
Total reflections	71114 (6494)	86834 (8769)	117298 (5856)
Unique reflections	53731 (2729)	42252 (4240)	23031 (1835)
Multiplicity	1.3 (1.3)	2.1 (2.1)	5.1 (3.0)
Completeness (%)	96.09 (95.22)	92.9 (93.4)	97.0 (79.2)
$I/\sigma(I)$	9.08 (1.00)	9.6 (1.3)	14.7 (0.9)
R-merge (%)	4.37 (60.75)	5.35 (63.4)	9.48 (134)
R-meas (%)	6.18 (85.91)	7.30 (86.4)	10.55 (162)
R-pim (%)	4.37 (60.75)	4.93 (58.4)	4.53 (89.6)
$CC_{1/2}$	0.997 (0.512)	0.998 (0.698)	0.998 (0.291)
Refinement			
Reflections	27443 (2727)	42252 (4239)	22908 (1835)
R-free	1297 (143)	2064 (183)	1170 (91)
R-work	19.33 (31.83)	21.23 (34.81)	22.25 (38.54)
R-free	23.34 (34.94)	26.95 (41.28)	25.05 (42.36)
Number of non-hydrogen atoms	2810	11219	3752
macromolecules	2574	11170	3746
ligands	32	12	
water	204	37	6
Protein residues	332	1427	484
r.m.s. deviation			
Bond lengths (Å)	0.005	0.003	0.002
Bond angles (º)	0.63	0.63	0.53
Ramachandran plot			
Favored (%)	99.09	96.87	96.22
Allowed (%)	0.91	3.13	3.78
Outliers (%)	0.00	0.00	0.00
Average B-factor	39.61	94.59	72.39
macromolecules	39.07	94.68	72.43
ligands	54.65	108.25	-
solvent	43.99	61.93	45.67

Statistics for the highest-resolution shell are shown in parentheses.

Supplementary Table 3. Oligonucleotide primers used in this study.

Primers	Company	Forward sequence	Reverse sequence
**FlhA**			
FlhA$^{C\text{-linker}}$	Sigma-Aldrich	5'-GGCGCCCATATGCGTGGTCGTGAAGAAAAA GC-3'	5'-CGTATGACCGCAACGATTGGTGGCAAATGACT CGAGG-3'
FlhA^{C}	Sigma-Aldrich	5'-GCCCATATGGAAGACTCACTGGGCATGGAAG-3'	5'-CGTATGACCGCAACGATTGGTGGCAAATGACT CGAGG-3'
FlhA^{C} I440A	Sigma-Aldrich	5'-GTTGGCTGGCAGCTAATCCGGGTAC-3'	5'-GTACCCGGATTAGCTGCCAGCCAAC-3'
FlhA^{C} D456V	Sigma-Aldrich	5'-GGGTGAAAAAAACCGTTGTGCCGGCATT TGGTCTGGACGC-3'	5'-GCGTCCAGACCAAATGCCGGCACAACGGTTTT TTCACCC-3'
FlhA^{C} F459A	Sigma-Aldrich	5'-GAAAAAACCGTTGATCCGGCAGCCGGT CTGGACGCTATTTG-3'	5'-CAAATAGCGTCCAGACCGGCTGCCGGA TCAACGGTTTTTTC-3'
FlhA^{C} L461A	Sigma-Aldrich	5'-GATCCGGCATTTGGTGCCGACGCTATT TG -3'	5'-CAAATAGCGTCGGCACCAAATGCCGGATC-3'
FlhA^{C} V482K	Sigma-Aldrich	5'-CAAATTCAGGGCTTCACGGTCAAAGAA GCAAGTACCGTTG-3'	5'-CAACGGTACTTGCTTCTTTGACCGTGAAGCCCT GAATTTG-3'
FlhA^{C} T490M	Sigma-Aldrich	5'-CAAGTACCGTTGTCGCTATGCATCTGAACCAC -3'	5'-GTGGTTCAGATGCATAGCGACAACGGTACTTG-3'
FlhA^{C} S637C		5'-CAGGAAGCACTGTGCCGCCAGGAAATGCT-3'	5'-AGCATTTCCTGGCGGCACAGTGCTTCCTG-3'
FlhA^{C} E640K	Sigma-Aldrich	5'-CAAACCCAGGAAGCACTGAGCCGCCAG AAAATGCTGGGTG-3'	5'-CACCCAGCATTTTCTGGCGGCTCAGTGCTT CCTGGGTTTG-3'
FlhA^{C} E640K/ M641G/L642G	Sigma-Aldrich	5'-CAGAAAGGCGGTGGTGCACCGCCG GTCCTGCTGGTGAATC-3'	5'-ACCGCCTTTCTGGCGGCTCAGTGCTTC CTGGGTTTG-3'
**FliT**			
FliT	Sigma-Aldrich	5'-GCCCATATGACCTCAACGGTGGAATTTATC-3'	5'-ATCCTCGAGATCCTCACGACGCA-3'
FliT Q26C	Sigma-Aldrich	5'-TGGAACTGGCTTGCCGTGGTGAATGGGATC-3'	5'-GATCCCATTCACCACGGCAAGCCAGTTCCAG-3'
FliT I93D	Sigma-Aldrich	5'- GAACTGAGCTCTCTGGACGGCCAGTCAACCC GCCAAAAATC-3'	5'-GATTTTTGGCGGGTTGACTGGCCGTCCAGAGA GCTCAGTTC-3'
FliT R98D	Sigma-Aldrich	5'- GATTGGCCAGTCAACCGACCAAAAATCGCTGA ATAATG-3'	5'-CATTATTCAGCGATTTTTGGTCGGTTGACTGGC CAATC-3'
FliT L102D	Sigma-Aldrich	5'- CGCCAAAAATCGGACAATAATGCCTATGGTCG C-3'	5'-GCGACCATAGGCATTATTGTCCGATTTTTGGC G-3'
FliT Y106G	Sigma-Aldrich	5'-CGCTGAATAATGCCGGTGGTCGCCTGTCG-3'	5'-CGACAGGCGACCACCGGCATTATTCAGCG-3'
**FliD**			
FliD	Sigma-Aldrich	5'-CAGGGCGCCCATATGGCTTCAATTTCATC-3'	5'-GCCCTCGAGTTAGGATTTATTCATCGCGGTG-3'
FliD^{C}	Sigma-Aldrich	5'-GCCCATATGTCCATCGACGAAACCGTTG-3'	5'-GCCCTCGAGTTAGGATTTATTCATCGCGGTG-3'
FliD Q441C	Sigma-Aldrich	5'- GCAGTTTACGTGCCTGGATACCATG-3'	5'-CATGGTATCCAGGCACGTAAACTGC-3'
**FliS**			
FliS	Sigma-Aldrich	5'-GCCCATATGTACACGGCGTCAGGTAT-3'	5'-GGAGCTCGGATCCGCGGCTTTCTTGGAATG-3'
FliS Y10A	Sigma-Aldrich	5'- GTATTAAGGCGGCCGCCCAGGTCTC-3'	5'-GAGACCTGGGCGGCCGCCTTAATAC-3'
FliS V13G	Sigma-Aldrich	5'-GTATGCCCAGGGTTCTGTTGAATC-3'	5'-GATTCAACAGAACCCTGGGCATAC-3'
**FliC**			
FliC	Sigma-Aldrich	5'-GCCCATATGATGGCACAAGTCATTAATAC AAACAGCC-3'	5'-TCCCTCGAGTCGCGCAATAATGAAAGCACG-3'
FliC^{C}	Sigma-Aldrich	5'- GGTACCGCAAGTGGGGCAGGCGGTTCTGAG GGAGGGGGCAGCGAAGGAGGTACGTCTGGAG CAACCGAGGACTCTGACTATGCC-3'	5'-TCCCTCGAGTCGCGCAATAATGAAAGCACG-3'
**FlgN**			
FlgN	Sigma-Aldrich	5'-GCCCATATGACCCGCCTGAGCGAAATC-3'	5'-GGATCCTTAGATACTAATCTTTTTACCGCCGC-3'
FlgN Y122G	Sigma-Aldrich	5'-CATCAAGAACCGACCCTGGGTGGTGCAGA-3'	5'-GTCTGCACCACCCAGGGTCGGTTCTTGATG-3'
